# A Review of the User Fees Policy for Primary Healthcare Consultations in Botswana: Problems With Effective Planning, Implementation and Evaluation

**DOI:** 10.34172/ijhpm.2021.141

**Published:** 2021-10-09

**Authors:** Vincent Pagiwa, Alan Shiell, Simon Barraclough, Onalenna Seitio-Kgokgwe

**Affiliations:** ^1^Okavango Research Institute, University of Botswana, Maun, Botswana.; ^2^School of Psychology and Public Health, La Trobe University, Melbourne, VIC, Australia.; ^3^Institute of Development Management, Gaborone, Botswana.

**Keywords:** Primary Healthcare, User Fees, Policy Implementation, Organizational Capacity, Botswana

## Abstract

**Background:** The Government of Botswana introduced user-fees for primary healthcare consultations in 1975. The policy has remained in place since then, although the fee has remained largely unaltered despite rising inflation. Early reviews of the policy pointed to problems in its implementation, but there has been no evaluation in the past 20 years. The aim of this study was to review the policy to assess whether documented issues with its implementation have been addressed.

**Methods:** This qualitative study involved interviews with 32 key informants: 18 policy-makers and 14 front-line revenue collectors. Data were analysed thematically using a template approach with constructs from an established organizational capacity assessment framework used as predetermined categories to guide data collection and analysis.

**Results:** Limited administrative and management capacity has been a major hindrance to effective implementation of the policy. The lack of infrastructure for effective revenue collection led to misappropriation of funds. Lack of clear guidelines for health facilities on how to implement the policy generated interdepartmental conflicts. Study participants believed the current policy was unlikely to be cost-effective since the cost of collecting fees probably exceeded the revenue it generated.

**Conclusion:** If the Botswana Government persists with the policy then it needs to improve organizational capacity to collect and manage revenues efficiently. However, policy thinking since the turn of the century has turned away from user-charges in healthcare as they impede the move towards universal access. It is timely therefore to consider alternative financing approaches that are more effective and a more equitable means of paying for healthcare.

## Background

**Key Messages**

** Implications for policy makers**

- It is vital periodically to evaluate policy and its implementation to ensure the policy continues to meet its stated objectives.
- If the Government of Botswana wishes to continue charging patients in primary care, it needs to re-assert the purpose of the policy and invest in the management and information systems necessary for its effective and efficient implementation.
- However, with the shift in policy thinking away from user-fees, and the growing recognition of the desirability of removing financial barriers to primary health, it is timely to reconsider the policy and whether alternative methods of financing healthcare are more consistent with current objectives for universal coverage.

** Implications for the public**

Policies on healthcare funding should be effectively planned, justified and evaluated. Without periodic evaluation, there is a danger that policies continue to be implemented even when they no longer achieve their original purpose, or that purpose is no longer valid. This study suggests that the benefits of collecting payments for primary healthcare consultations in Botswana are outweighed by the costs, and the stated purpose of the original policy - to sustain health system financing – has not been achieved. The users of the healthcare system would probably benefit from new ways of thinking about how the system should be funded.

In Botswana, responsibility for financing and managing public healthcare is vested with the Ministry of Health and Wellness (MoHW).^1^ The Ministry is a major provider of health services, which it does through a range of health facilities and management structures.^1^ It also plays a stewardship role, with District Health Management Teams, coordinating service provision at district and local levels, and it is responsible for the formulation of policies, regulations and norms, and guidelines for health services provision by public, non-profit and private healthcare providers.^1^ There is also an active private sector comprising private practitioners, and health services funded by the mining industry, non-governmental organisations and faith-based bodies.

 The Government of Botswana is the largest funder of health services, contributing over 60% of total health spending.^2-4^ Much of the remainder is paid for by private industry and donor agencies.^3-6^ A small proportion (less than 6% of the total) comes from out-of-pocket spending.^3,4^

 Similar to a number of African countries at the time, user fees were introduced in relation to primary healthcare consultations in 1975 in an effort to offset costs and raise revenue.^7,8^ For Botswana citizens and permanent residents, the fee in 2016 stood at P5.00 (Botswana Pula) (US$0.45). This covered unlimited consultations and access to services for three months. Non-citizens and private patients who used public healthcare services were charged P80.00 (US$7.50) for each consultation, plus extra charges for each service provided.

 Several problems have been reported in the management of this policy. Fees have remained largely unchanged despite rising prices. They have been increased only twice in the history of the policy, neither time in line with inflation. Other reported problems included high collection costs, misappropriation of fee revenues by health workers and inadequate policy and administrative support.^7,9,10^ Although the policy has been in existence for close to 50 years, there is limited understanding of the extent to which these documented challenges have been addressed. This paper examines the extent to which the challenges facing the user fee policy in Botswana have been addressed in the 20 years since it was last assessed.

## Methods

###  Study Design, Sampling and Data Collection

 This was a cross-sectional, qualitative study. Data were collected through semi-structured interviews with informants from MoHW departments, heads of referral hospitals, mission hospitals and District Health Management Teams, and front-line revenue collectors at two government hospitals and two mission hospitals. Interviews took place between February and October 2016. We used purposive sampling to select the participants for the study. Interviewed participants had worked in the health system of Botswana and had experience with implementing the user fees policy or were stakeholders involved in formulating or implementing the policy.

 Informants were recruited through formal letters and emails. Permission to approach front-line revenue collectors was first sought through the heads of their organizations before letters were sent to each collector inviting them to participate in the study. Invitations were sent to 50 participants in total: 20 policy-makers and 30 revenue collectors. Participants granted informed written consent before being interviewed. Interviews were conducted in English, which is the official language in the public sector in Botswana, and translation was not necessary. The average length of the interviews was 45 minutes.

 An interview guide developed specifically for this study was used in the data collection process. Interviews were conducted by the first author who was a PhD candidate. Data saturation was reached after interviews with 18 policy-makers and 14 revenue collectors. The interviews took place at each participant’s workstation and at a time chosen by the participant. With the participant’s permission, each interview was audio-recorded, and field notes were taken for non-verbal cues. The transcribed interviews were uploaded into Nvivo 11 software for coding and analysis.

###  Data Analysis

 The interview data were analysed thematically, using a template approach.^11-13^ Unlike grounded analysis where themes are identified inductively from the data, template analysis is a deductive approach that starts with a pre-specified set of codes, based in our case on the literature. There is a large body of evidence examining the assessment of organizational capacity to implement policy^14-20^ and our analytic approach was informed by this work. We relied most heavily on the framework developed by the United States Agency for International Development Capable Partners Botswana project.^19^ This framework was developed to analyse the capacity of non-governmental organisations working in HIV prevention.^19^ It has six constructs, five of which we regarded as especially pertinent to this review. These five constructs were: (*i*) governance and leadership; (*ii*) human resource management; (*iii*) sustainability; (*iv*) monitoring and evaluation; and (*v*) finance. The sixth construct related specifically to HIV/AIDs and was not used in this work.

 After transcribing the interviews in full, the first author (VP) read through the transcripts to familiarise himself with the data and to consider whether the template we had developed seemed suitable. He then began the process of coding the data, looking first for content that could be categorised under one or more of the five prespecified themes, and second for content that might require a new theme, not yet included in the template. In the event the template covered all the themes we identified in the data and no new themes were added. Quotations that were illustrative of each theme, were then highlighted. Next, the themes and the suggested quotations were reviewed and ratified for use in the paper in discussion between the first author and his thesis supervisors (AS and SB). The source of each of the quotations that we used was given a unique identification code for the purposes of the study.

## Results

 In reporting our results, we discuss each theme in the template in turn, using quotations from the participants to illustrate the issues raised. As stated, English is the official language of the public sector in Botswana, but our informants were not native English speakers. We cite the quotes here verbatim, including any grammatical errors, but annotate them where there would otherwise be loss of meaning.

###  Governance and Leadership

 The user fees policy is managed by the MoHW in the Department of Corporate Services, through the Finance and Accounts Unit. The implementation of the policy is delegated to health facilities. Although there was a clear leadership structure relating to the implementation of the policy, participants reported problems with governance and leadership that hampered effective revenue collection.

####  “Laissez Faire” Attitude From the MoHW

 The first set of problems arose from the “hands-off” leadership style adopted by the MoHW. Participants from health facilities viewed the MoHW staff as uninvolved and withdrawn from the implementation of the user fees policy. They said that the Ministry failed to provide detailed implementation guidelines and lacked the commitment to effect changes in operations. For example, there was no central direction from the Ministry about budgeting. Health facilities were free to forecast their budgets if they desired and to collect what they could. An informant from one facility reported:

*“We collect what is forthcoming. There is no obligation imposed to the facilities to say they should have a budget at a particular time, the collection will depend on their ability to collect as a facility*”(KIDP 1).

 Health facilities came up with different approaches to collect fees and document revenues. Some set targets for themselves and worked to meet the targets, while others saw no need to set targets. As two informants from different health facilities reported:

*“As an institution, we have a yearly target, which is also divided into monthly and quarterly targets. Our target does increase year after year. We increase by the percentage which we have over collected with*”(PMKI 1).

*“We do not have a target, we just collect. We cannot put a target since we are not sure how many people will come and who will pay. Maybe we will only see elderly people and children who are exempted from paying*”(HAK KI 1).

####  Conflicting Policies 

 The Botswana Government, through the National Health Policy, is committed to achieving universal health coverage and to providing services to people regardless of their ability to pay. This objective created a conflict with the user fees policy, which was exploited by some patients who were aware that they did not need to pay:

*“The same government says every person who comes and cannot pay for whatever reason, they still should be treated. In as far as it is a policy in cost recovery, there is a non-enforceable policy which renders the whole exercise a bit void. They come and they do not pay and if we do not treat them it will be a legal case against us..*.* Young working group *(referring to those who are able to pay)* come and while in the waiting area will be busy pressing their expensive gadgets*,* and then when it come to the time of payment, they refuse to pay P5.00” *(KAH KI 2).

*“They are aware they can come without money … the politicians say nobody should be sent back just because they do not have money*”(PMKI 2).

 The lack of political commitment to the scheme undermined the ability and morale of revenue collectors:

*“The system itself is politically motivated, so it is difficult for us to force people to pay. Actually, we have headache when it comes to collecting these fees. If then there were no restricting factors such as politicians and lack of policies, I would say the system could serve its purpose*”(KAH KI).

*“Our politicians should also buy in on what the ministry is trying to do, we can be able to do something*”(PMKI 1).

####  Inter-departmental Conflicts

 According to the implementation protocols, patients attending hospitals should be received by the registry department, which then refers them to the revenue office for payment before their consultation and treatment. In some cases, patients would be seen by the clinical staff prior to paying the fee. In these instances, the medical staff were supposed to refer the patient back to the revenue office to settle their payments. Participants reported that medical and allied health staff were unwilling to ask patients to settle their payments, since this was outside their job description:

*“Nobody understands what it is that is to be collected, more especially doctors and nurses. Even if they are asked to refer patients for payments, they don’t do that. The revenue departments are the ones who usually battle with people to pay but the medical staff is not supportive. People come to see them, but if they do not care about fees attached to those services then we are not going anywhere*”(KIDPS).

 At district health centres and clinics, nurses and other healthcare workers who used to collect user fees demanded extra work allowances since they thought collecting user fees was outside their job description. According to some participants, the government relieved nurses and other allied health staff of the responsibility to collect fees. Consequently, no collection of user fees occurred at health centres and clinics where there were no dedicated collectors:

*“There is no collection at all in clinics since nurses were refrained from collecting. We realized the need to relieve nurses in clinics from collecting because there were issues of overworking and misappropriation of funds due to multitasking*”(KICS).

###  Human Resource Management

####  Staffing

 A shortage of revenue collectors was another factor that led to poor revenue collection. Healthcare services were centralized in 2002 and revenue collectors were absorbed into local government.^21^ This reduced the number of revenue collection departments and the number of revenue collectors. This affected the scheduling of shifts and meant that revenue collection was sometimes halted, especially at night and weekends. As one stakeholder explained:

*“The major challenge is that we do not have enough revenue collectors, we stopped collection at night due to these shortages. We realized at night there are few patients coming to the hospital and mostly are emergency cases which are exempted from paying*”(KICS).

 The reduction in staff numbers placed stress on the remaining revenue collectors. Some started to take sick leave, while others began to dodge calls to take up shifts for their colleagues:

*“The job is a very straining job, and the revenue collectors are not enough. This ends up straining them and we have many sick leaves coming in*” (BLH KI 2).

*“The challenge is revenue collectors get tired and opt to get sick leave. This affects collection as there will be no replacement for the sick person. Sometimes I try to call other revenue collectors to cover for the sick person, so when they see it is work phone number, they either don’t answer, or they switch off their phone*”(HLB KI 2).

####  Developing Human Resources

 It is mandatory for every government employee in Botswana to attend an induction course within the first year of employment to be introduced to government systems.^22^ However, some revenue collectors reported not having been given the opportunity to attend an induction course. The lack of in-service training meant that people had to learn on the job, which was not always ideal:

*“There was no formal training since we were employed. We just started working with our high school qualification. We learn in the job by receiving training from our colleagues we find in the service”*(KAH RC 1).

*“We were not trained, we just got to learn in the job by the help of the old staff. No formal training was offered to us*”(NRH RC3).

*“The revenue collectors are not trained enough and no refresher courses as an incentive, there is not even a budget for training our staff, most of the funds for training are always towards the medical staff than administration*”(BLH KI 2).

 The lack of training, and lack of support more generally from their managers, seemed to contribute to a sense of being under-valued among revenue collectors. Combined with their low pay, this rendered the system vulnerable to fraud. As two informants explained:

*“We do not receive enough support more especially when we have questions or suggesting ways of improving our working conditions. I think just because we are collecting P5.00 they do not think there is value in developing our department*”(KAH RC1).

*“Our revenue collectors are not well trained and are paid less and that has led to issues in mishandling funds*”(NRH KI 2).

####  Incentives and Motivation

 Some participants were concerned that the revenue that was collected from user fees was transferred to the Government Treasury, and it was not obvious whether any of this income returned to the health system. Thus, there was no incentive, either locally or in the Ministry, to put any effort into fee collection. The MoHW leadership did not see the user fees policy as a priority. There was no plan to improve the revenue collection system, nor any motivation to evaluate the policy. Participants explained that remitting all revenue to the Treasury was a disincentive for active collection:

*“Personally, I think we haven’t done anything to say we have a system in place. There is nothing documented about this system, no billing systems, money goes to the general revenue pool, so this is just an extra job imposed on us*”(KIHODP).

*“We collect money, but it ends up being taken by the government. If we could be given a certain percentage from the collection as an incentive, I will say we can continue with it*”(KIDP).

###  Finance

####  Misappropriation of Collected Funds

 There were problems in some facilities concerning the management of collected fees. These included misappropriation of funds by the revenue collectors and difficulties transporting money between the collection points and the government revenue offices. There was a concern in some health facilities about the time it took revenue collectors to remit the funds that they had accrued and there were reports of revenue collectors taking money generated from user fees for their personal use, albeit with the intention to repay later. Health facilities usually accounted for the revenue income through the Government Accounting and Budgeting system (GABS), but some still relied on their own manual accounts. Misappropriation of funds was reported more commonly at hospitals still using a manual collection system:

*“The system is open to money being mishandled. We have issues where revenue collectors borrow themselves government money and sometimes fail to return it when the money is demanded. The government finance policy suggests that when they collect an amount of P 1000, they should remit, but they usually stay until they accumulate large sums of money; that’s where problems arise of money shortages*”(KIDHP).

*“There are incidences of fees being mishandled since we still use the manual system. Five years ago, there was a misappropriation of funds by about 3 to 4 revenue collectors… the revenue collectors will falsely write the receipts and misappropriate the funds*”(KAH KI 2).

####  Security of Funds

 The lack of facilities to keep funds and transport them securely was raised as one of the concerns in the management of user fees revenue. Some hospitals lacked strong rooms and lockable safes in which to keep the collected revenue. This was reported by revenue collectors as risky because it was susceptible to theft. Revenue collectors reported having to use vehicles that did not have lockable storage boxes to keep money safe during transportation. This lack of security also jeopardized the safety of revenue collectors who feared they might be robbed:

*“There is no safety at all, we are afraid sometimes we might be attacked and robbed of the money collected. We sometimes work weekends and after hours when the accounts staff have knocked off. Therefore, we have to leave cash where it is not safe and the following day, we are expected to remit enough cash*”(HLB RC 1).

 The lack of facilities to manage and securely store funds was also an issue of concern for the Treasury auditors as they considered it to be against the finance standards of Botswana. One participant had this to say:

*“In other facilities, we were forced to halt collection because we did not comply with finance standards. We still use old facilities which when they were built it was not taken in consideration that finances will be collected, therefore, when they come for audit, they find that we use simple rooms with no safe for money and they will recommend we stop collecting because the facility is not conducive*”(KIDP 2).

####  Information and Technology Systems

 Key informants stated that when the MoHW instituted a new Integrated Patient Management System in 2004, the idea was to incorporate the billing of patients through the same system, but this has still not happened. Payments that have been collected can be accounted for through the GABS, but this does not integrate with the patient management system. In the absence of a computerised billing system, it was easy for patients to miss being charged whilst still receiving treatment. As two informants reported:

*“There is a system called Integrated Patient Management System, which we had thought will capture the medical data of the patients *(and)* at the same time cost the services provided to the patient. However*,* we have not been able to put that into practice, the system is only used for medical data rather than financial. Currently collection is entered into the government GABS system, which is linked directly to the general revenue pool*”(KICS).

*“Currently there is no billing system in public facilities… Since the government has no billing system, it is difficult to claim money from patients even those with medical aid schemes*.* The problem we have is that people still think services are for free. There is also a phenomenon that we care for life first, that makes it difficult for the system to take its course*”(KIHODP).

###  Monitoring and Evaluation

####  Data Management

 The GABS has other problems.^23^ It is not especially efficient and when data have been released, they were often outdated and not aligned with management cycles:

*“The major challenge we have is our information system, the data from facilities is not adequate to help us plan. If you do not (have) adequate information you can’t come up with effective solutions*.* There is a backlog of data or information, like currently we rely on data for 2010 while we are supposed to be using 2016 data. We cannot plan effectively with 2010 data for 2016 and beyond. I think the major evaluation that need to be done is on our information systems*”(KIDP 1).

*“It is difficult to make an evaluation of this system because of the data base we have. We usually rely on the data from Statistics Botswana but there are a lot of loopholes and the data is always outdated. The other issue is that the fees go to the government revenue pool where it is difficult to separate it from the rest of other sources of revenue. We really cannot say there is much done to evaluate this system*”(KIDPS).

####  Monitoring Performance at Facility Level

 In addition to data management issues, local management were reluctant to carry out monitoring activities because of the lack of clear policy objectives. One informant pointed out that:

*“Since it is not clear what the objectives are for this policy … no measurable objectives clearly stipulated to what extent the cost recovery should impact on the government expenditure. The purpose of the user-fees introduction is not clearly outlined, so it is very difficult to measure if the system has been able to achieve its intended objectives*” *** (*** KIDP 1)

 Furthermore, revenue collectors reported a lack of support from their supervisors when they raised ideas that they believed could help improve the operation of the user fees system. As one collector explained:

*“I would not say they support us; they hardly listen to our inputs even when we have ideas that can help improve collection of fees*”(NRH RC 5).

###  Sustainability

####  Abolition of the User Fees Scheme

 Participants reported that the user fee policy was not a sustainable way of raising revenue as it was costly to operate and would be difficult to improve. In addition, there was no costing and billing system that could enable public health facilities to claim payments from private health insurance for patients willing to pay through their insurance cover. Key informants were of the view that the user fees policy should be abolished, and the Government should establish a prepayment system through health insurance. As one of them suggested:

*“Like the National Health Policy has recommended, I am in support that the user fees should be abolished. A prepayment system will be ideal since people will not feel a burden when they must seek for healthcare services. The current system is not value adding at all*”(KIDP 1).

## Discussion

 The study examined the implementation of user fees for primary healthcare in Botswana using an established tool for assessing organizational capacity for policy implementation. Before discussing the results, several limitations should be noted. First, it proved difficult to find documentary evidence on the initial design and purpose of the policy, and on the results of past evaluations. In one case, this involved tracking down a report in the private collection of a retired bureaucrat. Much of the history of the policy is now lost in time. We have also relied heavily on the views of current health system employees. Resource constraints prevented us from seeking out the views of past health system employees or current staff in Treasury or Finance departments. Our initial intention of supporting the qualitative data reported here with a quantitative analysis of revenue collection over time was hindered by incomplete and out of date data systems.

 Whatever the situation when the policy was first introduced, it is evident from our results that successive governments have failed to respond to previously documented shortcomings in the capacity to implement the policy. The problems reported to us were largely the same as those found in earlier evaluations. There has been a lack of both leadership and investment in the infrastructure needed to make the policy work. Mixed messages about the direction of the policy have undermined local support and willingness to collect fees. The absence of effective monitoring and enforcement systems has led to inconsistent implementation, as well as misuse and misappropriation of revenue. All of this has fuelled conflict and undermined relationships between clinical and revenue staff.

 The challenges that we have documented here are similar to those reported elsewhere. Revenue systems that are complicated and poorly administered can lead to corruption and mismanagement of funds.^24^ Such abuse is found in other Sub-Saharan countries, including Kenya^25,26^ and Tanzania,^27^ and this erodes confidence in user fee schemes.^28-30^ The lack of enforcement mechanisms, inadequate staffing, lack of resources to collect fees, weak administration and the lack of political will, have all been documented in other developing countries.^31-34^

 The user-fees policy in Botswana was introduced in the mid-1970s as part of an effort to relieve pressure on public sector budgets.^8^ However, as a consequence of the shortcomings in its implementation that we have described here, the user-fees policy appears not to make economic sense. It has not reduced the costs of healthcare because patients know they can continue to use services and yet easily avoid paying the fee, and it has not raised revenue, since it appears to cost more to administer than the income it generates. If the Government wishes to persist with the user-fees policy, then it needs to consider the deficiencies we have noted here, and to invest in building the organizational capacity and infrastructure needed to implement the policy properly and to monitor and evaluate its performance so that continuing issues can be quickly identified and addressed.

 Alternatively, now is as good a time as any for the Government to reflect on the objectives of its health policy and the role that user fees can (and cannot) play in achieving its ends. Despite being under increasing financial pressure from the mounting burden of communicable disease, most notably HIV/AIDs and weaknesses in the economy,^35^ Botswana is committed to achieving universal access to health services.^36,37^ At issue is whether user fees intended to reduce financial pressure on the budget are compatible with the drive towards universal access. If fees are imposed across the board, then they likely deter low-income groups from seeking timely care.^38-40^ This undermines universal access, and the evidence suggests this will lead to worse health outcomes and higher costs in future.^25,38,39,41^ If fee-exemptions are allowed to protect the needy, then the revenue-raising ability of the policy is diminished. Furthermore, since there are costs associated with revenue collection, including the policing of exemptions, the more exemptions are allowed the more likely it is that the costs will exceed the value of the revenue generated.

## Conclusion

 Our findings suggest that for effective implementation of the policy there is a need to reconsider its aims, invigorate its central leadership, align incentives faced by different players in the system, and enhance the organizational capacity locally to collect and safely remit revenues. On the other hand, health policy thinking has shifted in the 45 years since the user-charges policy was first introduced. Support for this method of financing healthcare has fallen because of mounting evidence of the adverse effects it has on access to healthcare and the household finances of people living in poverty.^40,42-45^ International agencies such as the World Health Organization

 (WHO) and the World Bank are now urging countries to reduce financial barriers to primary care as part of a move towards universal access to healthcare.^46,47^ Thus, it is timely for the Government of Botswana to reflect on its experience with user-fees, to consider whether the policy is consistent with its aim to promote universal access, and to assess the feasibility of alternative approaches to financing health care, such as mandatory prepayment methods, that are more supportive of this objective.^48^

## Acknowledgements

 The authors are grateful to the La Tobe University Graduate Research School for funding the project. Authors also want to thank the MoHW in Botswana for their participation in this study.

## Ethical issues

 This study received ethical clearance from La Trobe University Human Research Ethics Committee and Botswana’s MoHW Research Ethics Unit.

## Competing interests

 The authors declare that they have no financial or personal relationship(s) that may have inappropriately influenced them in writing this article.

## Authors’ contributions

 VP participated in the conception and design of the study, acquisition of data, analysis and interpretation of data, drafting of the manuscript, obtaining funding, critical revision of the manuscript for important intellectual content and administrative support. AS participated in the conception and design of the study, analysis and interpretation of data, drafting of the manuscript, obtaining funding, critical revision of the manuscript for important intellectual content, supervision and administrative, technical, or material support. SB participated in the conception and design of the study, analysis and interpretation of data, drafting of the manuscript, obtaining funding, critical revision of the manuscript for important intellectual content, supervision and administrative, technical, or material support. OSK participated in the analysis and interpretation of data, drafting of the manuscript, critical revision of the manuscript for important intellectual content and administrative, technical, or material support.

## Disclaimer

 The views and opinions expressed in this article are those of the authors and do not necessarily reflect the official or position of any affiliated agency of the authors.

## Funding

 The study was funded by the Graduate Research School at La Trobe University, Melbourne, VIC, Australia.
